# Supplementary material for: Anti-spike antibody response to natural SARS-CoV-2 infection in the general population
Source: Nat Commun. 2021 Oct 29;12:6250. doi: 10.1038/s41467-021-26479-2 (PMC8556331; doi:10.1038/s41467-021-26479-2)
Supplement: Supplementary file 1 — Supplementary Information [file 41467_2021_26479_MOESM1_ESM.pdf]

Supplementary information for

Anti-spike antibody response to natural SARS-CoV-2 infection in the general population

Jia Wei, Philippa C. Matthews, Nicole Stoesser, Thomas Maddox, Luke Lorenzi, Ruth Studley, John I Bell, John N Newton, Jeremy Farrar, Ian Diamond, Emma Rourke, Alison Howarth, Brian D. Marsden, Sarah Hoosdally, E Yvonne Jones, David I Stuart, Derrick W .Crook, Tim E. A. Peto, Koen B. Pouwels, A. Sarah Walker, David W. Eyre and the COVID-19 Infection Survey team

Contents

- **Supplementary Tables 1-9**
- **Supplementary Figures 1-11**

	Total (N=7256)	Class 1 'seroconverted' (N=4683)	Class 2 'possible late/reinfection' (N=831)	Class 3 'seronegative non-responders' (N=1742)	p value (all)	P value (2 vs 1)	P value (3 vs 1)
**Percentage**	100%	64.5%	11.5%	24.0%			
**Symptoms & Ct value**					< 0.001	< 0.001	< 0.001
**No self-reported symptoms & Ct>=30**	1779 (24.5%)	132 (2.8%)	528 (63.5%)	1119 (64.2%)			
**No self-reported symptom & Ct<30**	1287 (17.7%)	912 (19.5%)	122 (14.7%)	253 (14.5%)			
**Self-reported symptoms & Ct>=30**	1057 (14.6%)	641 (13.7%)	128 (15.4%)	288 (16.5%)			
**Self-reported symptom & Ct<30**	3133 (43.2%)	2998 (64.0%)	53 (6.4%)	82 (4.7%)			
**Have >=2 positive swabs in the infection episode**					< 0.001	< 0.001	< 0.001
**No**	4285 (59.1%)	1975 (42.2%)	628 (75.6%)	1682 (96.6%)			
**Yes**	2971 (40.9%)	2708 (57.8%)	203 (24.4%)	60 (3.4%)			
**Days between first and last positive swab if >=2 positive swabs**					< 0.001	0.002	< 0.001
**Median**	14	14	18	20			
**IQR**	7, 26	7, 26	10, 28	10, 29			
**Have negative study swab before index positive**					< 0.001	< 0.001	< 0.001
**Yes**	6203 (85.5%)	4032 (86.1%)	603 (72.6%)	1568 (90.0%)			
**No**	1053 (14.5%)	651 (13.9%)	228 (27.4%)	174 (10.0%)			
**Days between previous negative and index positive**					< 0.001	< 0.001	< 0.001
**Median**	26	25	29	25			
**IQR**	12, 35	13, 34	25, 60	7, 29			
**Have national testing programme positive included within infection episode**					< 0.001	< 0.001	< 0.001
**No**	5675 (78.2%)	3210 (68.5%)	756 (91.0%)	1709 (98.1%)			
**Yes**	1581 (21.8%)	1473 (31.5%)	75 (9.0%)	33 (1.9%)			
**National testing programme positive before study index positive**					< 0.001	< 0.001	< 0.001
**No**	6053 (83.4%)	3570 (76.2%)	764 (91.9%)	1719 (98.7%)			
**Yes**	1203 (16.6%)	1113 (23.8%)	67 (8.1%)	23 (1.3%)			
**Days between national testing programme positive and study index positive**					< 0.001	< 0.001	< 0.001
**Median**	7	6	15	16			
**IQR**	3, 14	3, 13	10, 24	10, 23			

Supplementary Table 1. Additional characteristics of classes identified from latent class mixed models for 7,256 participants infected with SARS-CoV-2. Main characteristics among classes are presented in **Table 1**, and continuous variables are presented graphically in **Supplementary Fig. 2**. Continuous variables were compared using Kruskall-Wallis tests, and categorical variables were compared using one-sided Chi-squared tests.

	Class 1<br>Seroconverted	Class 2<br>Possible late/reinfection			Class 3<br>Seronegative		
		OR	95%CI	p-value	OR	95%CI	p-value
**Age**	ref	See **Fig. 2**					
**Sex**							
**Female**		1(ref)			1(ref)		
**Male**		0.98	0.81-1.18	0.8	0.88	0.74-1.04	0.1
**Ethnicity**							
**White**		1(ref)			1(ref)		
**Non-white**		1.03	0.75-1.40	0.9	0.80	0.60-1.08	0.2
**Report having long-term health conditions**							
**No**		1(ref)			1(ref)		
**Yes**		0.82	0.64-1.04	0.1	1.10	0.90-1.35	0.4
**Report working in patient facing healthcare**							
**No**		1(ref)			1(ref)		
**Yes**		1.02	0.52-1.96	1	0.39	0.18-0.83	**0.01**
**Ct value (one unit higher)**		1.35	1.32-1.39	**<0.001**	1.33	1.31-1.36	**<0.001**
**Report having symptoms**							
**No symptoms**		1(ref)			1(ref)		
**Other symptoms**		0.09	0.07-0.13	**<0.001**	0.23	0.19-0.29	**<0.001**
**Classic symptoms**		0.07	0.05-0.09	**<0.001**	0.07	0.06-0.09	**<0.001**
**Have >=2 positive swabs in the infection episode**							
**No**		1(ref)			1(ref)		
**Yes**		1.13	0.80-1.60	0.5	0.08	0.05-0.13	**<0.001**
**Days between first and last positive (1 day longer)**		0.99	0.98-1.00	0.1	1.00	0.98-1.02	0.8

Supplementary Table 2. Odds ratio with 95% confidence intervals from multinomial logistic regression, using Class 1 as the reference. See **Fig. 2** for effect of age and other factors on the probability scale. The 95% confidence intervals are calculated by prediction ± 1.96*standard error of the prediction; two-sided Wald p values are shown. Bold numbers indicate significant p values <0.05.

	Class 1 'seroconverted'	Class 3 (A: Using all data): 'seronegative non responders'			Class 3 (B: Conditioning on Ct ≤32 and ≥2 gene positivity)		
		OR	95%CI	p-value	OR	95%CI	p-value
**Age**	ref			**<0.001**			**<0.001**
**Sex (Male vs Female)**		0.84	0.75-0.96	**0.008**	0.87	0.73-1.04	0.1
**Ethnicity (Non-White vs White)**		0.75	0.60-0.94	**0.01**	0.88	0.63-1.20	0.4
**Report having long-term health conditions (Yes vs No)**		1.15	0.99-1.34	0.06	0.97	0.78-1.21	0.8
**Report working in patient facing healthcare (Yes vs No)**		0.42	0.22-0.75	**0.005**	0.44	0.15-1.02	0.09
**Cough (Yes vs No)**		0.20	0.15-0.25	**<0.001**	0.24	0.16-0.35	**<0.001**
**Loss of smell (Yes vs No)**		0.21	0.13-0.33	**<0.001**	0.34	0.17-0.62	**<0.001**
**Fever (Yes vs No)**		0.42	0.29-0.60	**<0.001**	0.51	0.29-0.84	**0.01**
**Loss of taste (Yes vs No)**		0.53	0.35-0.79	**0.002**	0.51	0.27-0.89	**0.03**
**Fatigue/weakness (Yes vs No)**		0.58	0.44-0.75	**<0.001**	0.69	0.46-1.01	0.06
**Headache (Yes vs No)**		0.69	0.54-0.87	**0.003**	0.54	0.37-0.79	**0.002**
**Sore throat (Yes vs No)**		0.69	0.52-0.91	**0.01**	0.59	0.37-0.91	**0.02**
**Myalgia (Yes vs No)**		0.75	0.55-1.02	0.07	0.70	0.43-1.09	0.1
**Nausea/Vomiting (Yes vs No)**		0.93	0.59-1.42	0.7	1.25	0.66-2.25	0.5
**Diarrhoea (Yes vs No)**		1.06	0.66-1.68	0.8	0.88	0.39-1.79	0.7
**Abdominal pain (Yes vs No)**		1.07	0.62-1.78	0.8	0.98	0.41-2.07	1
**Shortness of breath (Yes vs No)**		1.39	0.97-1.95	0.07	1.60	0.97-2.57	0.06

Supplementary Table 3. Odds ratio with 95% confidence intervals from logistic regression comparing seronegative vs seroconverting (Class 3 vs Class 1) using demographic factors and individual symptoms that would be available without a positive test result. (A) Using all data from Class 3 (N=1,742) vs Class 1 (N=4,683). **(B)** Restricting Class 3 to those with Ct value ≤32 and ≥2 genes detected (N=595) to decrease the impact of potential false positive swab tests. Age was fitted using natural cubic spline with one internal knot placed at 50 years and two boundary knots at 20, 80 years. Effect of age is presented in **Supplementary Fig. 4**. The 95% confidence intervals are calculated by prediction ± 1.96*standard error of the prediction; two-sided Wald p values are shown. Bold numbers indicate significant p values <0.05. Odds ratios and 95% confidence intervals are visually presented in **Fig. 3**.

	Class 1 'seroconverted'	Class 3 (A: Using all data): 'seronegative non responders'			Class 3 (B: Conditioning on Ct ≤32 and ≥2 gene positivity)		
		OR	95%CI	p-value	OR	95%CI	p-value
**Age**	ref			**<0.001**			**<0.001**
**Sex (Male vs Female)**		0.84	0.73-0.97	**0.02**	0.85	0.69-1.04	0.1
**Ethnicity (Non-White vs White)**		1.26	0.98-1.62	0.07	1.17	0.82-1.69	0.4
**Report having long-term health conditions (Yes vs No)**		1.19	1.00-1.43	0.05	1.06	0.81-1.38	0.7
**Report working in patient facing healthcare (Yes vs No)**		0.42	0.21-0.84	**0.01**	0.35	0.11-1.13	0.08
**Cough (Yes vs No)**		0.18	0.13-0.24	**<0.001**	0.20	0.13-0.33	**<0.001**
**Loss of smell (Yes vs No)**		0.21	0.12-0.36	**<0.001**	0.31	0.15-0.66	**0.002**
**Fever (Yes vs No)**		0.38	0.24-0.58	**<0.001**	0.40	0.21-0.77	**0.006**
**Loss of taste (Yes vs No)**		0.45	0.28-0.72	**<0.001**	0.47	0.23-0.95	**0.03**
**Fatigue/weakness (Yes vs No)**		0.58	0.43-0.80	**<0.001**	0.70	0.44-1.11	0.1
**Headache (Yes vs No)**		0.64	0.48-0.86	**0.003**	0.60	0.39-0.94	**0.03**
**Sore throat (Yes vs No)**		0.63	0.46-0.88	**0.006**	0.55	0.32-0.92	**0.02**
**Myalgia (Yes vs No)**		0.83	0.59-1.18	0.3	0.66	0.38-1.15	0.1
**Nausea/Vomiting (Yes vs No)**		0.84	0.50-1.43	0.5	1.11	0.52-2.35	0.8
**Diarrhoea (Yes vs No)**		0.95	0.53-1.72	0.9	0.74	0.27-2.01	0.6
**Abdominal pain (Yes vs No)**		0.96	0.50-1.83	0.9	0.96	0.35-2.63	0.9
**Shortness of breath (Yes vs No)**		1.39	0.93-2.10	0.1	1.39	0.76-2.53	0.3
**Angina (Yes vs No)**		0.96	0.43-2.14	0.9	1.04	0.38-2.82	0.9
**Asthma (Yes vs No)**		0.84	0.66-1.06	0.1	0.93	0.67-1.30	0.7
**Atrial fibrillation (Yes vs No)**		1.07	0.59-1.94	0.8	1.04	0.46-2.35	0.9
**Cancer (Yes vs No)**		1.07	0.80-1.43	0.6	1.03	0.69-1.56	0.9
**Chronic kidney disease (Yes vs No)**		1.13	0.70-1.84	0.6	0.94	0.46-1.94	0.9
**Chronic liver disease (Yes vs No)**		0.88	0.39-1.99	0.8	0.92	0.29-2.94	0.9
**COPD (Yes vs No)**		0.69	0.40-1.17	0.2	1.37	0.72-2.60	0.3
**Coronary heart disease (Yes vs No)**		0.99	0.40-2.43	1	1.19	0.39-3.66	0.8
**Frailty (Yes vs No)**		0.64	0.41-1.01	0.05	0.48	0.24-0.98	**0.04**
**Heart failure (Yes vs No)**		0.97	0.37-2.60	1	0.93	0.26-3.35	0.9
**Hypertension (Yes vs No)**		0.87	0.64-1.17	0.3	0.81	0.52-1.24	0.3
**Myocardial infarction (Yes vs No)**		1.45	0.61-3.48	0.4	1.21	0.37-3.98	0.8
**Osteoporosis (Yes vs No)**		2.00	1.07-3.73	**0.03**	1.33	0.52-3.42	0.6
**Peripheral arterial disease (Yes vs No)**		0.66	0.32-1.37	0.3	0.63	0.22-1.81	0.4
**Rheumatoid arthritis (Yes vs No)**		0.82	0.32-2.12	0.7	0.89	0.23-3.53	0.9

**Stroke (Yes vs No)**	1.11	0.47-2.62	0.8	0.53	0.11-2.59	0.4
**Transient ischaemic attack (Yes vs No)**	0.77	0.32-1.84	0.6	1.50	0.50-4.50	0.5
**Type 1 diabetes (Yes vs No)**	3.06	0.95-9.85	0.06	3.10	0.52-18.55	0.2
**Type 2 diabetes (Yes vs No)**	1.29	0.76-2.18	0.3	0.94	0.43-2.05	0.9
**Overweight (BMI 25 to <30 kg/m^2) (Yes vs No)**	0.73	0.61-0.88	**<0.001**	0.80	0.61-1.05	0.1
**Obese (BMI ≥30 kg/m^2) (Yes vs No)**	0.84	0.68-1.03	0.1	0.88	0.65-1.19	0.4
**Antihypertensive medication (Yes vs No)**	1.00	0.76-1.33	1	0.95	0.64-1.42	0.8
**Diabetes medication (Yes vs No)**	0.47	0.26-0.86	**0.01**	0.45	0.17-1.15	0.09
**Corticosteroids (Yes vs No)**	1.53	0.87-2.71	0.1	0.83	0.34-2.04	0.7
**Immunosuppressants (Yes vs No)**	0.79	0.31-2.05	0.6	0.96	0.24-3.81	1

Supplementary Table 4. Odds ratio with 95% confidence intervals from a logistic regression model comparing seronegative vs seroconverting participants (Class 3 vs Class 1) using demographic factors, individual symptoms, and comorbidities. (A) Using all data from Class 3 (N=1,383) vs Class 1 (N=4,032). **(B)** Restricting Class 3 to those with Ct value ≤32 and ≥2 genes detected (N=487) to decrease the impact of potential false positive swab tests. Age was fitted using natural cubic spline with one internal knot placed at 50 years and two boundary knots at 20, 80 years. Comorbidities were obtained by linkage to the General Practice Extraction Service (GPES) Data for Pandemic Planning and Research and Hospital Episode Statistics (HES) via the NHS number. The 95% confidence intervals are calculated by prediction ± 1.96*standard error of the prediction; two-sided Wald p values are shown. Bold numbers indicate significant p values <0.05.

		Peak level (ng ml^{-1})			Half-life (days)		
		Posterior mean	95% CrI		Posterior mean	95% CrI	
**Model from 56 days after index positive**	Censored at 800 (base case)	203	190	210	184	163	210
	Censored at 500	194	189	200	187	168	209
	Censored at 400	189	183	194	192	172	215
**Model from 28 days after index positive**	Censored at 800	225	213	232	189	171	211
	Censored at 500	216	210	222	184	168	202
	Censored at 400	209	204	215	187	172	204
**Model from 84 days after index positive**	Censored at 800	177	164	184	225	182	287
	Censored at 500	168	163	175	233	192	290
	Censored at 400	164	158	170	242	199	305

Supplementary Table 5. Sensitivity analysis using different starting point (28, 84 days) and different censoring threshold (400, 500 ng ml^{-1}) for anti-spike IgG trajectory modelling (peak and half-life estimation).

		Estimate	Est.Error	95%Crl		Rhat	Bulk_ESS	Tail_ESS
**Baseline**	Intercept	7.6636	0.0247	7.6149	7.7119	1.0020	2802	4046
	time	-0.0054	0.0003	-0.0061	-0.0048	1.0004	8113	6588
	sigma	0.5165	0.0117	0.4943	0.5401	1.0045	261	856
	sd(Intercept)	1.0406	0.0250	0.9925	1.0895	1.0010	651	1953
	sd(time)	0.0043	0.0009	0.0021	0.0058	1.0087	137	211
	cor(Intercept,time)	-0.2576	0.0976	-0.4166	-0.0467	1.0015	1365	1047
**Age**	Intercept	7.6405	0.0244	7.5932	7.6881	1.0016	2299	4221
	time	-0.0049	0.0004	-0.0057	-0.0042	1.0006	6927	6056
	age	0.1194	0.0163	0.0871	0.1509	1.0044	1988	3551
	time:age	-0.0001	0.0002	-0.0005	0.0004	1.0015	5853	6699
	sigma	0.5164	0.0117	0.4935	0.5398	1.0151	304	798
	sd(Intercept)	1.0222	0.0239	0.9762	1.0695	1.0078	645	2515
	sd(time)	0.0043	0.0009	0.0023	0.0058	1.0355	147	186
	cor(Intercept,time)	-0.2409	0.0943	-0.4008	-0.0338	1.0057	920	906
**Sex**	Intercept	7.6376	0.0339	7.5700	7.7040	1.0012	2330	4247
	time	-0.0044	0.0005	-0.0053	-0.0034	1.0005	5928	6931
	sex	0.0531	0.0488	-0.0429	0.1486	1.0007	2225	3689
	time:sex	-0.0022	0.0007	-0.0035	-0.0009	1.0005	6714	6566
	sigma	0.5179	0.0118	0.4954	0.5417	1.0218	224	421
	sd(Intercept)	1.0393	0.0246	0.9910	1.0879	1.0076	575	2035
	sd(time)	0.0041	0.0010	0.0015	0.0057	1.0448	119	113
	cor(Intercept,time)	-0.2615	0.1057	-0.4333	-0.0319	1.0069	1667	1136
**Ethnicity**	Intercept	7.6216	0.0259	7.5706	7.6722	1.0019	2110	3747
	time	-0.0053	0.0004	-0.0060	-0.0046	1.0008	5942	6477
	ethnicity	0.4344	0.0833	0.2660	0.5929	1.0005	2263	4012
	time:ethnicity	-0.0018	0.0011	-0.0039	0.0004	1.0001	5954	6204
	sigma	0.5163	0.0115	0.4937	0.5392	1.0052	267	1149
	sd(Intercept)	1.0340	0.0245	0.9880	1.0831	1.0038	626	2504
	sd(time)	0.0043	0.0009	0.0024	0.0059	1.0131	113	334
	cor(Intercept,time)	-0.2512	0.0927	-0.4074	-0.0427	1.0033	953	1222
**LTHC**	Intercept	7.6294	0.0276	7.5750	7.6833	1.0011	2125	4128
	time	-0.0054	0.0004	-0.0062	-0.0047	1.0004	7285	6868
	LTHC	0.1646	0.0630	0.0416	0.2879	1.0006	2121	3843
	time:LTHC	0.0002	0.0009	-0.0017	0.0020	1.0007	5814	6214
	sigma	0.5188	0.0119	0.4959	0.5429	1.0308	213	568
	sd(Intercept)	1.0347	0.0248	0.9870	1.0828	1.0145	484	842
	sd(time)	0.0040	0.0010	0.0014	0.0057	1.0409	122	238
	cor(Intercept,time)	-0.2406	0.1093	-0.4122	0.0049	1.0076	1159	747
**Ct value**	Intercept	7.6610	0.0247	7.6127	7.7098	1.0008	2161	4383
	time	-0.0055	0.0004	-0.0062	-0.0048	1.0001	7177	6326
	ct	0.0056	0.0040	-0.0024	0.0133	1.0015	2173	4200
	time:ct	0.0000	0.0001	-0.0001	0.0001	1.0002	6296	7132
	sigma	0.5170	0.0117	0.4953	0.5414	1.0088	217	293
	sd(Intercept)	1.0390	0.0244	0.9914	1.0869	1.0044	530	1126
	sd(time)	0.0042	0.0010	0.0016	0.0058	1.0200	108	168
	cor(Intercept,time)	-0.2540	0.1041	-0.4155	-0.0414	1.0059	947	507
**Symptom**	Intercept	7.6801	0.0543	7.5737	7.7884	1.0004	2481	4294
	time	-0.0066	0.0008	-0.0081	-0.0051	1.0002	6162	5701
	symptom	-0.0195	0.0608	-0.1395	0.0996	1.0012	2486	4307
	time:symptom	0.0014	0.0009	-0.0002	0.0031	1.0002	5915	6392
	sigma	0.5157	0.0126	0.4922	0.5411	1.0200	223	475
	sd(Intercept)	1.0407	0.0256	0.9900	1.0907	1.0118	428	1126
	sd(time)	0.0043	0.0010	0.0019	0.0060	1.0330	117	155
	cor(Intercept,time)	-0.2565	0.0982	-0.4161	-0.0412	1.0071	845	818
**Multivariable**	Intercept	7.5307	0.0603	7.4120	7.6482	1.0022	2235	3459
	time	-0.0045	0.0009	-0.0062	-0.0027	1.0003	6706	5743
	age	0.1336	0.0169	0.1011	0.1672	1.0001	2283	4077
	sex	0.0464	0.0488	-0.0510	0.1416	1.0009	2368	4226
	ethnicity	0.5297	0.0817	0.3720	0.6927	1.0006	2408	3995

	LTHC	0.0994	0.0613	-0.0178	0.2175	1.0009	2373	4184
	ct	0.0091	0.0040	0.0012	0.0168	1.0000	2178	3914
	symptom	0.0088	0.0593	-0.1065	0.1269	1.0028	2029	4134
	time:age	-0.0002	0.0002	-0.0007	0.0003	1.0001	6309	6334
	time:sex	-0.0021	0.0007	-0.0034	-0.0008	1.0002	7569	6581
	time:ethnicity	-0.0021	0.0011	-0.0042	0.0000	1.0003	6654	7010
	time:LTHC	0.0000	0.0009	-0.0018	0.0018	1.0004	7363	7377
	time:ct	0.0000	0.0001	-0.0001	0.0001	0.9999	7213	6771
	time:symptom	0.0009	0.0009	-0.0007	0.0027	1.0002	5384	6424
	sigma	0.5186	0.0122	0.4950	0.5431	1.0040	224	406
	sd(Intercept)	1.0064	0.0252	0.9576	1.0563	1.0038	385	931
	sd(time)	0.0038	0.0012	0.0008	0.0056	1.0160	131	171
	cor(Intercept,time)	-0.2162	0.1272	-0.4037	0.0933	1.0030	586	326

Supplementary Table 6. Model coefficients and MCMC diagnostics for the univariable and multivariable models. The reference category in the multivariable model is: 43-year-old, female, white ethnicity, no long-term health conditions, Ct value=22, and no self-reported symptoms.

		Multivariable model		
		Posterior mean	95% CrI	
**Baseline**	Peak level (Intercept)	178	151	195
	IgG half-life (slope)	256	168	439
**Age**	Peak level: 43 years (median)			
	IgG half-life: 43 years (median)			
	Change in peak level: per 10-year older	18*	14	23
	Change in half-life: per 10-year older	-12	-54	22
**Sex**	Peak level: Female			
	IgG half-life: Female			
	Change in peak level: Male	6	-6	18
	Change in half-life: Male	-92*	-236	-25
**Ethnicity**	Peak level: White			
	IgG half-life: White			
	Change in peak level: Non-white	78*	50	107
	Change in half-life: Non-white	-85	-223	5
**LTHC**	Peak level: No			
	IgG half-life: No			
	Change in peak level: Yes	13	-2	30
	Change in half-life: Yes	15	-115	227
**Ct value**	Peak level: 22 (median)			
	IgG half-life: 22 (median)			
	Change in peak level: per 1 unit higher	1*	0	2
	Change in half-life: per 1 unit higher	0	-9	9
**Symptom**	Peak level: No			
	IgG half-life: No			
	Change in peak level: Yes	1	-14	15
	Change in half-life: Yes	73	-76	236
**Time period**	Peak level: Pre-Alpha			
	IgG half-life: Pre-Alpha			
	Change in peak level: Alpha	12	-1	25
	Change in half-life: Alpha	-35	-153	46

Supplementary Table 7. Posterior mean and 95% credibility intervals for anti-spike IgG peak level (intercept) (ng ml^{-1}) and half-life (slope) (days) in multivariable model including the time period factor (pre-Alpha vs Alpha) in 3,271 participants in Class 1. Pre-Alpha period: before 16 Nov 2020; Alpha period: 17 Nov 2020-16 May 2021. Due to the short follow-up time in Delta period (after 17 May 2021), no Delta period participants are included. The reference category is: 43-year-old, female, white ethnicity, no long-term health conditions, Ct value=22, no self-reported symptoms, and in pre-Alpha period. * indicates where the multivariable 95% CrI excludes 0 (no effect).

		**Biphasic exponential model**						**Linear exponential model**					
**A**		**42 ng ml^{-1}**		**28 ng ml^{-1}**		**6 ng ml^{-1}**		**42 ng ml^{-1}**		**28 ng ml^{-1}**		**6 ng ml^{-1}**	
**Age**	**Sex&ethnicity**	**Estimate**	**95%CrI**	**Estimate**	**95%CrI**	**Estimate**	**95%CrI**	**Estimate**	**95%CrI**	**Estimate**	**95%CrI**	**Estimate**	**95%CrI**
**20**	White female	590	270-2176	762	402-2879	1430	817-5825	502	239-980	644	364-1232	1186	782-2051
	Non-white female	485	278-1138	593	362-1389	1012	640-2458	436	261-747	530	341-898	887	621-1470
	White male	407	207-758	520	309-952	955	639-1761	361	192-580	454	280-698	815	590-1180
	Non-white male	380	235-656	463	302-796	775	535-1341	347	220-526	417	281-619	686	504-980
**40**	White female	649	329-1859	817	463-2277	1466	888-4186	529	289-926	660	409-1117	1163	802-1909
	Non-white female	524	313-1155	630	389-1429	1040	670-2395	457	284-748	547	364-875	885	631-1406
	White male	449	251-778	562	350-936	988	687-1634	386	228-590	476	308-697	817	609-1122
	Non-white male	410	262-699	495	329-824	801	565-1365	366	240-536	433	299-627	693	522-972
**60**	White female	703	371-2654	869	482-3145	1500	871-5973	552	316-989	673	418-1196	1140	781-1966
	Non-white female	561	334-1456	667	407-1710	1070	669-2827	476	306-798	562	378-917	884	628-1448
	White male	490	286-923	600	376-1123	1017	685-1909	408	251-623	495	330-737	819	605-1158
	Non-white male	441	285-809	520	343-962	826	571-1532	385	258-565	448	317-651	699	525-1000
**80**	White female	755	374-7812	917	470-7812	1534	813-7812	571	335-1180	686	422-1385	1119	733-2056
	Non-white female	598	347-2267	703	411-2819	1097	650-4521	495	320-896	574	380-1023	882	613-1605
	White male	531	308-1348	638	385-1674	1050	655-2866	430	272-692	511	341-817	821	587-1276
	Non-white male	471	300-1017	551	357-1211	851	557-1920	400	275-618	464	327-712	704	515-1075
**B**		**42 ng ml^{-1}**		**28 ng ml^{-1}**		**6 ng ml^{-1}**		**42 ng ml^{-1}**		**28 ng ml^{-1}**		**6 ng ml^{-1}**	
**Group**	**Multiplier**	**Estimate**	**95%CrI**	**Estimate**	**95%CrI**	**Estimate**	**95%CrI**	**Estimate**	**95%CrI**	**Estimate**	**95%CrI**	**Estimate**	**95%CrI**
**60-year-old white male**	1	490	286-923	600	376-1123	1017	685-1909	408	251-623	495	330-737	819	605-1158
	2	301	118-616	412	221-797	830	548-1557	261	112-441	347	195-552	673	486-970
	3	191	57-426	301	118-616	720	467-1341	178	57-341	261	112-441	588	412-853
	5	71	57-247	162	57-391	581	361-1093	68	57-222	155	57-323	479	312-714
	10	56	57-69	56	57-164	394	202-747	58	57-75	58	57-161	333	183-530

**60-year-old white female**	1	700	369-2616	868	489-3290	1498	873-5864	551	317-990	674	425-1184	1138	776-1955
	2	418	141-1498	586	283-2035	1218	701-4483	344	131-660	463	241-842	930	625-1596
	3	253	57-907	418	141-1498	1051	594-3942	218	57-486	344	131-660	809	525-1406
	5	70	57-390	210	57-775	841	457-3124	64	57-305	186	57-450	653	404-1154
	10	56	57-64	56	57-209	553	255-1999	58	57-69	58	57-196	444	224-826

Supplementary Table 8. A) Posterior predicted days with 95% credibility interval from the start of infection to three anti-spike IgG thresholds (42, 28, and 6 ng ml^{-1}) by age (20, 40, 60, 80 years), sex, and ethnicity in the multivariable biphasic exponential model and linear exponential model. B) Posterior predicted days with 95% credibility interval from the start of infection to thresholds (42, 28, and 6 ng ml^{-1}) multiplied by 2, 3, 5, 10 in 60-year-old white male and female population in the multivariable biphasic exponential model and linear exponential model.

Model term	Priors
**Intercept**	normal (7.4, 2.8)
**Slope**	normal (0, 0.1)
**Coefficient for change in intercept (age)**	normal (0, 2)
**Coefficient for change in slope (age)**	normal (0, 0.1)
**Coefficient for change in intercept (sex)**	normal (0, 6)
**Coefficient for change in slope (sex)**	normal (0, 0.1)
**Coefficient for change in intercept (ethnicity)**	normal (0, 10)
**Coefficient for change in slope (ethnicity)**	normal (0, 0.2)
**Coefficient for change in intercept (long-term health condition)**	normal (0, 7)
**Coefficient for change in slope (long-term health condition)**	normal (0, 0.2)
**Coefficient for change in intercept (Ct)**	normal (0, 1)
**Coefficient for change in slope (Ct)**	normal (0, 0.1)
**Coefficient for change in intercept (symptom)**	normal (0, 6)
**Coefficient for change in slope (symptom)**	normal (0, 0.1)
**Random effect SD: intercept**	normal (0, 1)
**Random effect SD: slope**	normal (0, 0.01)
**Random effect intercept & slope covariance**	lkj_corr_cholesky (2)

Supplementary Table 9. Priors used in the Bayesian linear mixed interval censored models.

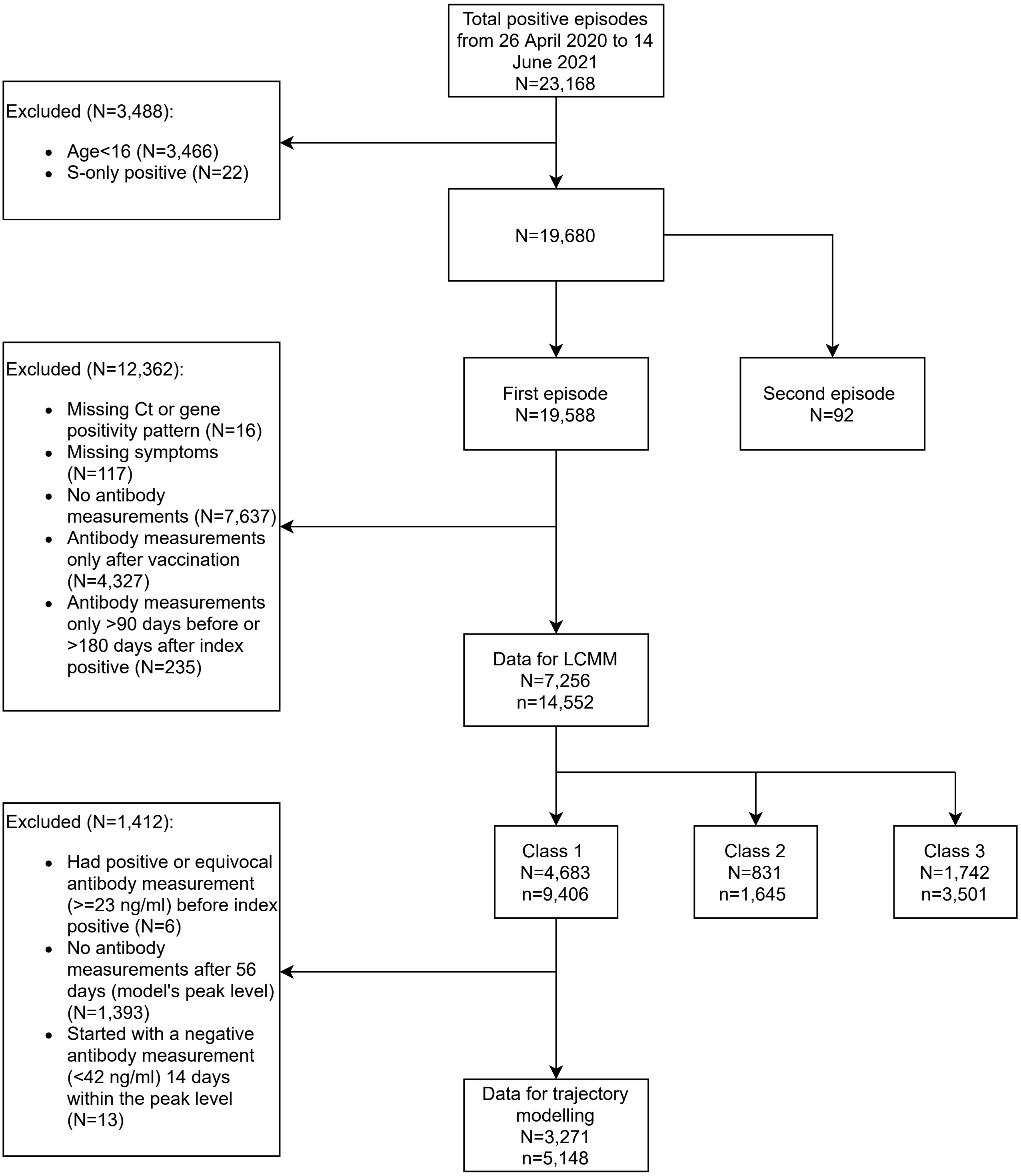

Supplementary Fig. 1. Flowchart of the study cohort. N represents the number of positive episodes/ participants, and n represents the number of antibody measurements. LCMM=latent class mixed model. Class 1='seroconverted in response to infection', Class 2='possible late/ reinfection', Class 3='seronegative non-responders', see **Fig. 1** and **Supplementary Fig. 3**.

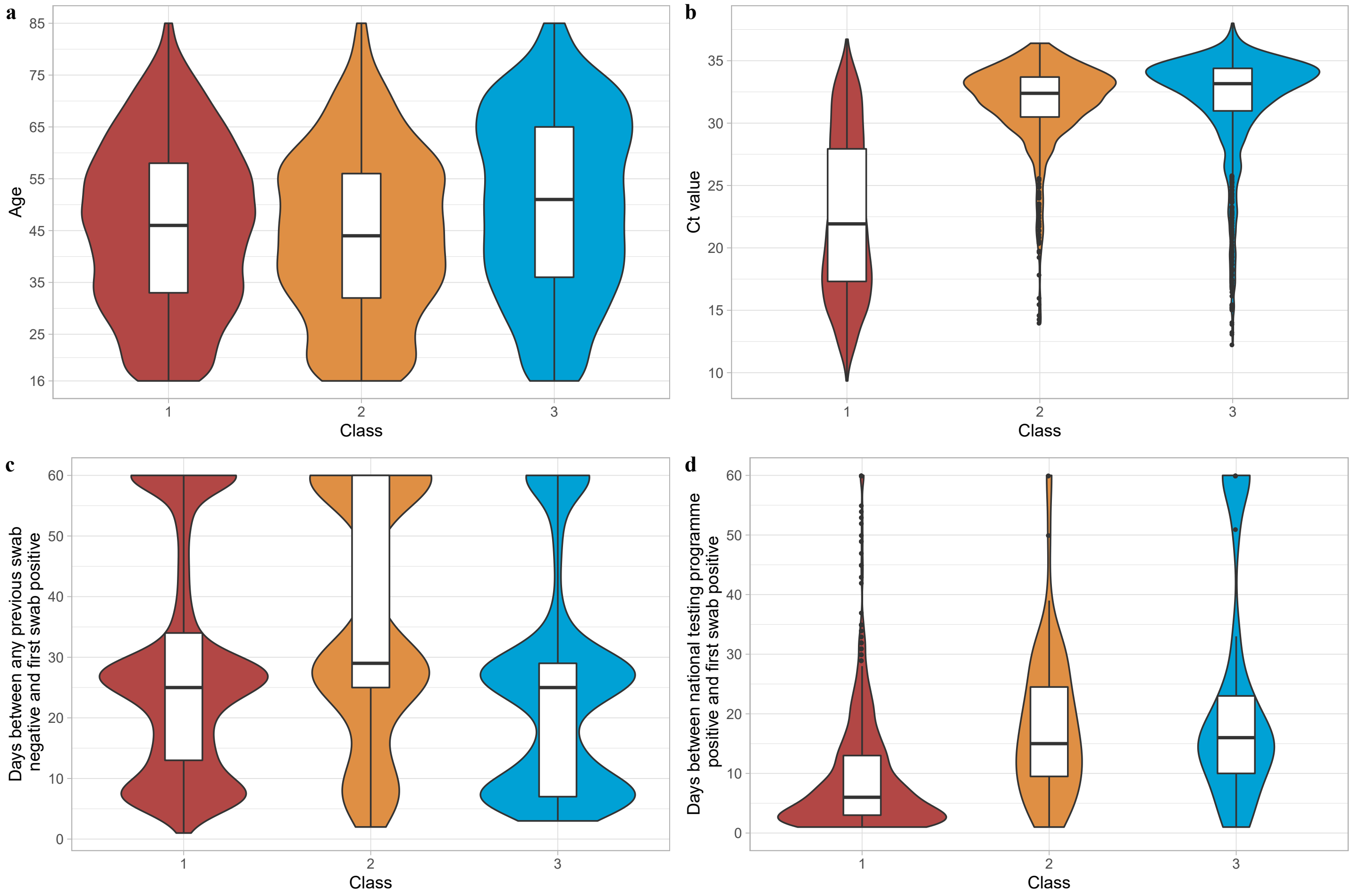

Supplementary Fig. 2. Distributions of continuous variables by classes identified from latent class mixed models. a, Participants' age. Participants in Class 3 were older than Class 1 and 2. **b**, Participants' minimum Ct value across the infection episode. Class 1 had a lower Ct value than Class 2 and 3. **c**, Days between any previous study swab negative test and the first swab positive test in the infection episode. More participants in Class 2 had a longer duration between previous negative swab and the index positive, supporting late detection. **d,** Days between any preceding national testing programme positive test and the first swab positive test (y-axis truncated at 60 days for visualisation). Class 1 had a shorter duration between the two tests than other two classes. Class 1='seroconverted in response to infection' (N=4,683, 64.5%), Class 2='possible late/reinfection' (N=831, 11.5%), Class 3='seronegative non-responders' (N=1,742, 24.0%). For the box and whisker inserts: centre line, median; box limits, upper and lower quartiles; whiskers, 1.5x interquartile range; points, outliers.

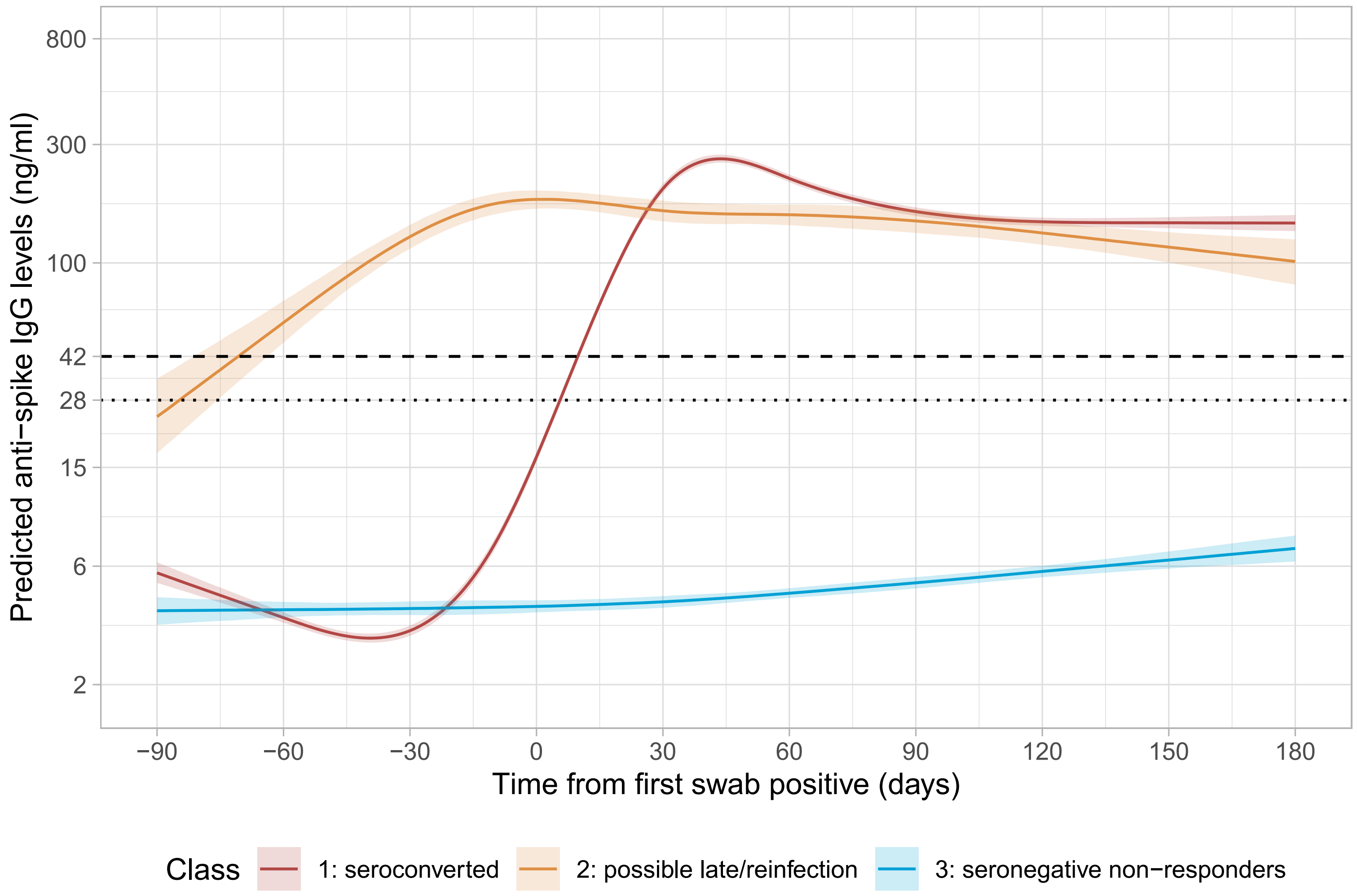

Supplementary Fig. 3. Predicted mean anti-spike IgG trajectories in participants with natural SARS-CoV-2 infection from latent class mixed models in 7,256 participants (with standard error of the prediction). Black dashed line indicates the assay threshold for IgG positivity (42 ng ml^{-1}) and the dotted line at 28 ng ml^{-1} indicates level associated with 50% protection against reinfection. The 95% confidence interval are calculated by a Monte Carlo approximation of the posterior distribution of the predicted values. Class 1='seroconverted in response to infection' (64.5%, n=4683), Class 2='possible late/reinfection' (11.5%,n=831), Class 3='seronegative non-responders' (24.0%, n=1742).

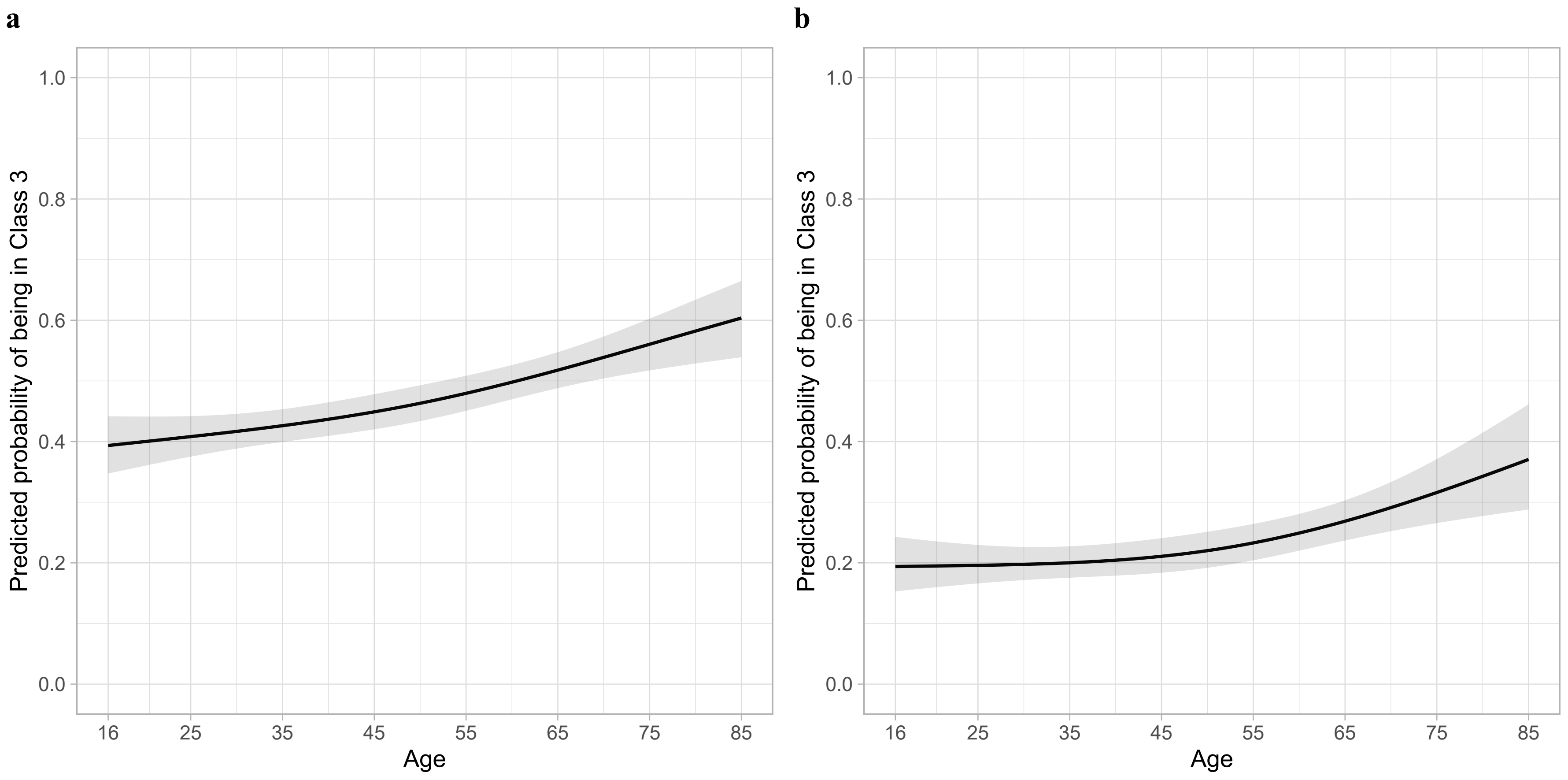

Supplementary Fig. 4. Predicted probability of being in Class 3 (seronegative) compared with Class 1 (seroconverted) by age. Plotted at the reference category for other variables (female, white ethnicity, no long-term health condition, not working in patient-facing healthcare, no self-reported symptoms of any kind). **a,** Using all data from Class 3 (N=1,742). **b**, Conditioning on Ct value ≤32 and ≥2 genes detected (N=595) to decrease the impact of potential false positive swab tests. Age was fitted using natural cubic spline with one internal knot placed at 50 years and two boundary knots at 20, 80 years. Full model's results are presented in **Fig. 3, Supplementary Table 3**. The 95% confidence intervals are calculated by prediction ± 1.96*standard error of the prediction.

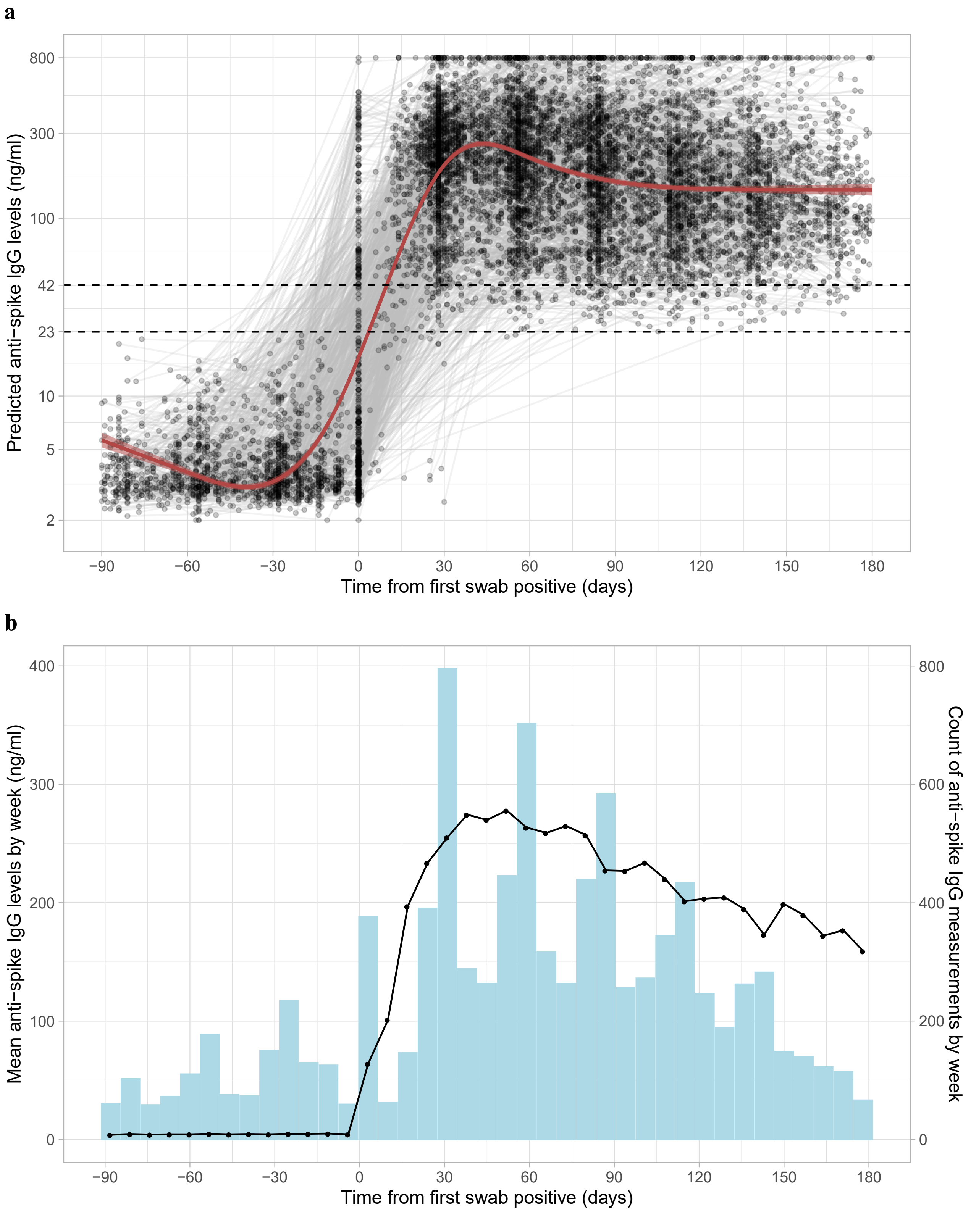

Supplementary Fig. 5. Determination of the 'peak level' for modelling anti-spike IgG trajectories. a, Class trajectory and individual trajectories for participants included in the model from Class 1. **b,** Mean anti-spike IgG levels and count of IgG measurements by week after index positive test. Around 56 days (8 weeks), the number of IgG measurements is high, and the mean IgG levels are close to the highest level.

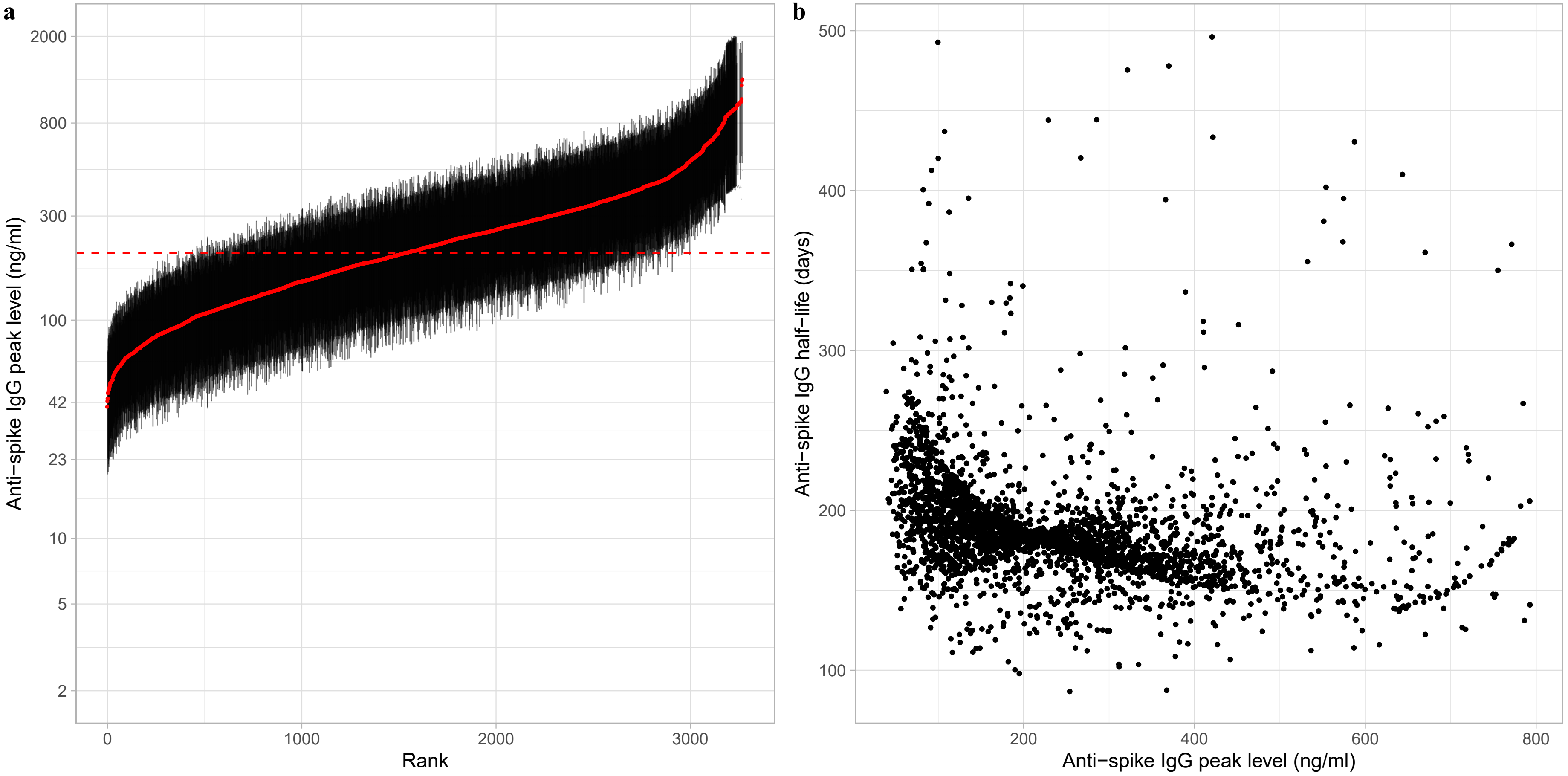

Supplementary Fig. 6. Posterior estimations for individual anti-spike IgG half-lives and peak level. a, Estimated peak anti-spike IgG level with 95% CrI for all participants (N=3,271) by rank. The red dot shows the mean level in each individual. The red dashed line shows the overall mean level (203 ng ml^{-1}). **b,** Relationship between estimated IgG peak levels with half-lives. X and Y axes are truncated at 800 and 500 for visualization (Two-sided Spearman correlation test. Spearman's rank coefficient=-0.5, p<0.0001).

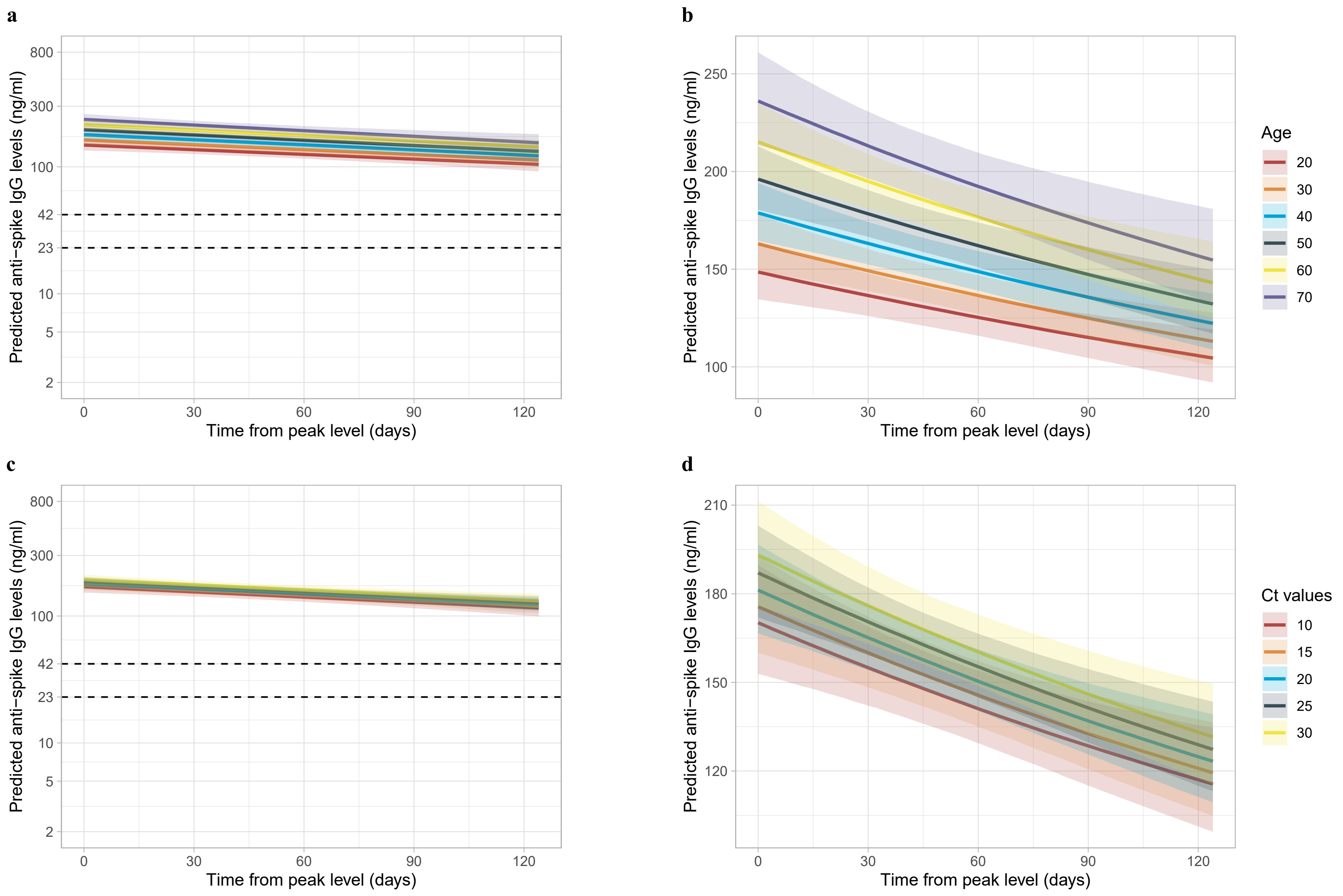

Supplementary Fig. 7. Posterior mean trajectories with 95% credibility intervals of anti-spike IgG after the peak level by age (a, b) and Ct values (c, d) in the multivariable model in 3,271 participants. Panel a and c show the relationship on the log10 scale. Black dashed line indicates the assay threshold for IgG positivity (42 ng ml^{-1}) and the dotted line at 28 ng ml^{-1} (indicates level associated with 50 % protection against reinfection). Panel b and d show the relationship in its original scale. Full model results are shown in **Table 2**.

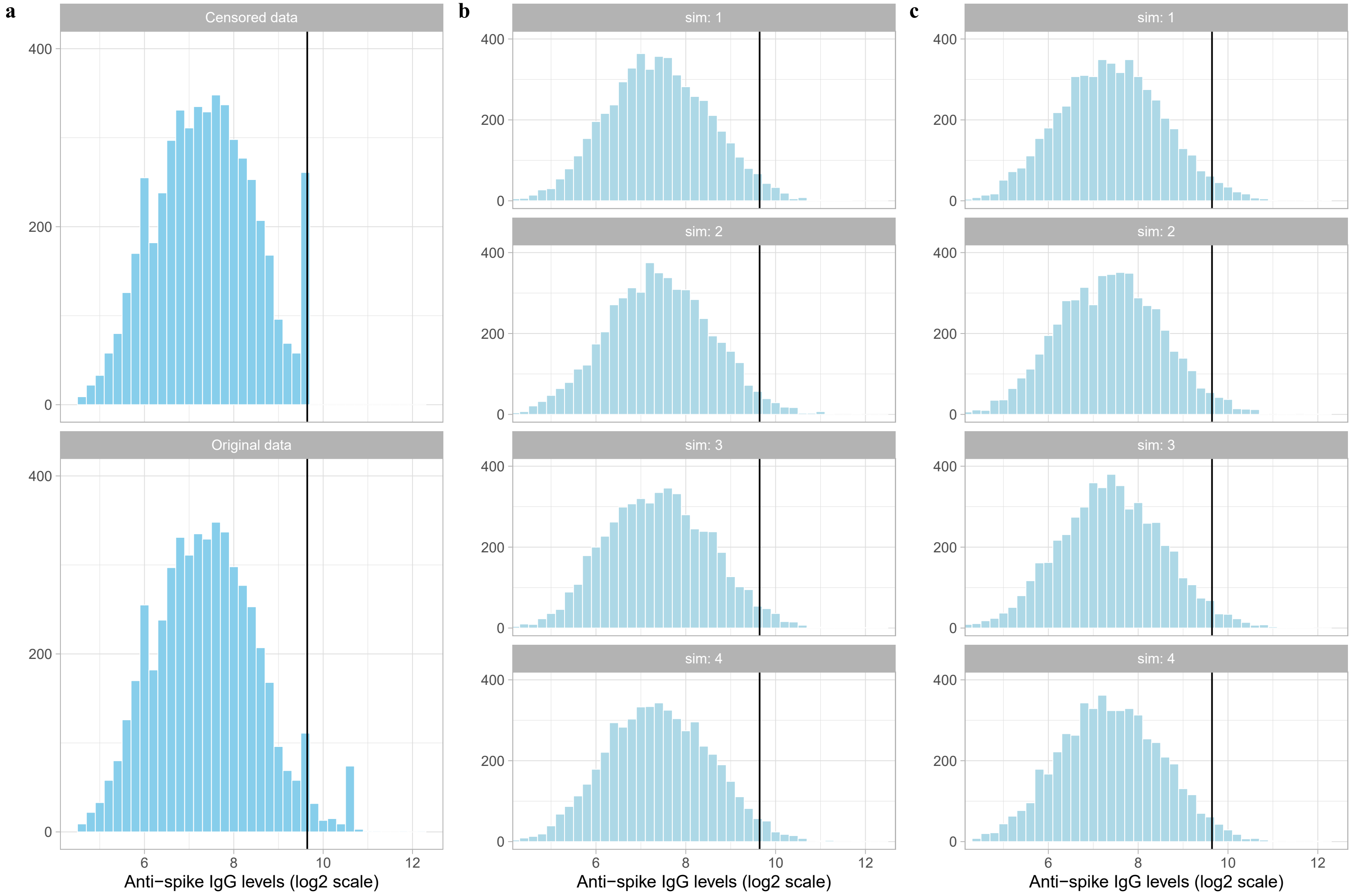

Supplementary Fig. 8. Posterior predictive check for the baseline model and the multivariable model. a, Distribution of the observed anti-spike IgG levels on log2 scale. **b,** Distribution of anti-spike IgG levels from four posterior simulated datasets for the baseline model. **c,** Distribution of anti-spike IgG levels from four posterior simulated datasets for the multivariable model.

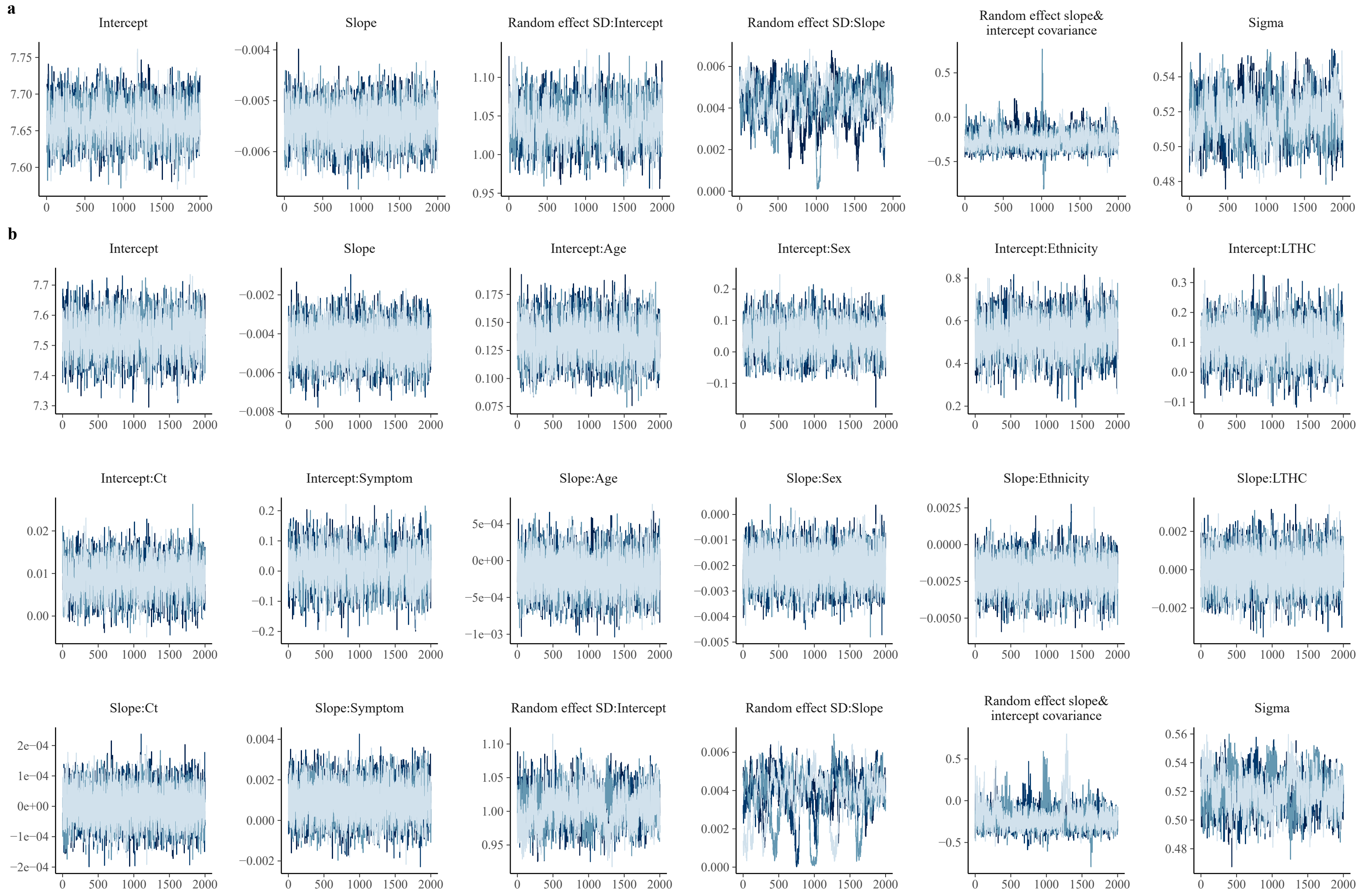

Supplementary Fig. 9. MCMC trace plots for assessing convergence of chains. a, Trace plots for the baseline model. **b,** Trace plots for the multinomial model.

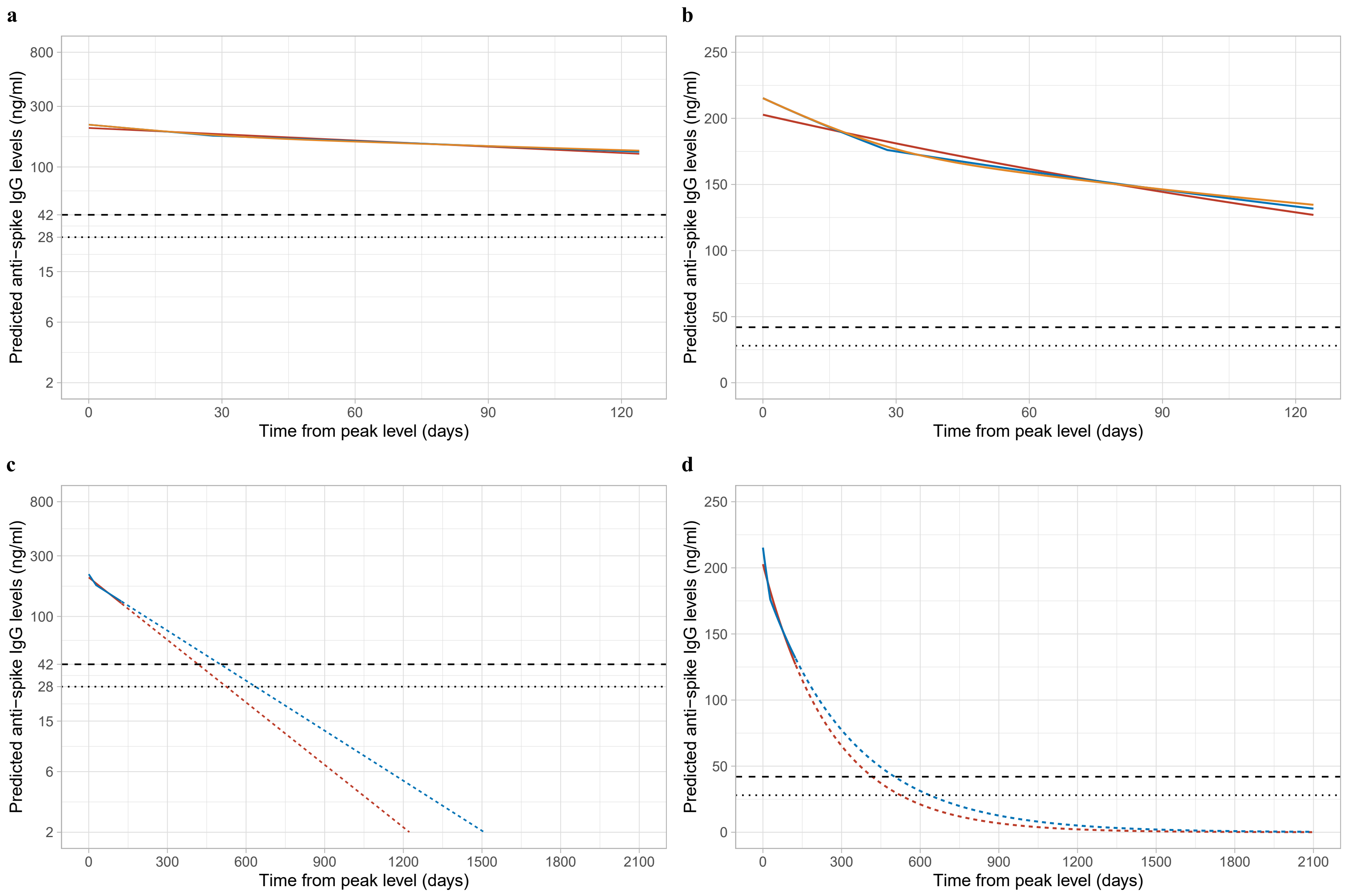

Supplementary Fig. 10. Comparison of anti-spike IgG decline in 3,271 participants among the model assuming a linear decline in log2 scale (red line), the biphasic exponential model (blue line), and the model using splines (orange line). For the biphasic model, knot is placed at 28 days. For the spline model, time is fitted using natural cubic splines with internal knots at 30, 70 and boundary knots at 5, 110. Panel a, b show the antibody trajectories from the three models within observed period (124 days). Panel c,d show the predicted trajectories from the linear and biphasic models over 2100 days (dotted line indicates the values are extrapolated). Panel a,c shows the trajectory on the log10 scale. Panel b,d shows the trajectory in its original scale. Black dashed line indicates the assay threshold for IgG positivity (42 ng ml^{-1}) and the dotted line at 28 ng ml^{-1} (indicates level associated with 50 % protection against reinfection). The spline model had the lowest LOOIC (11402), followed by the biphasic model (11594), and the linear model (11819).

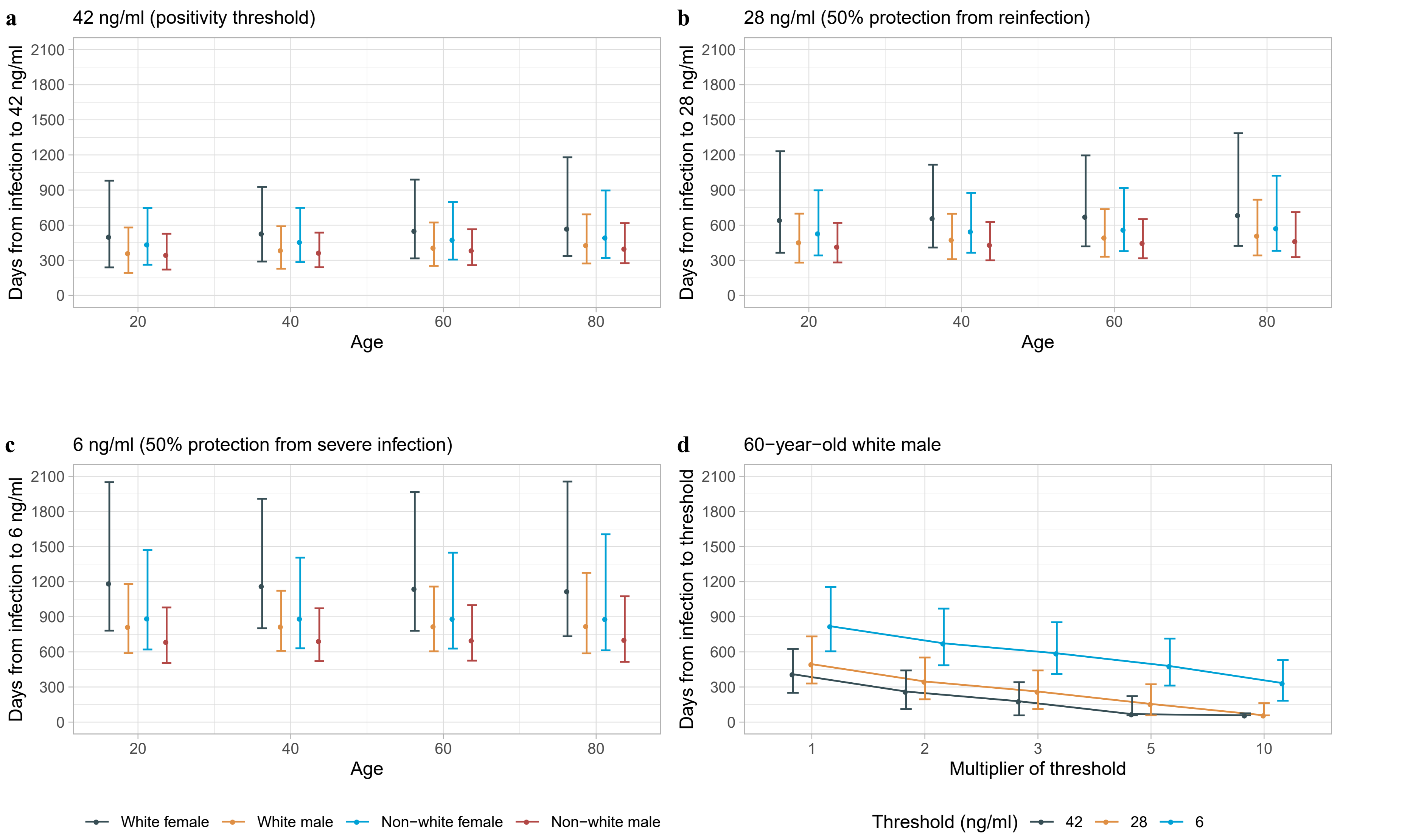

Supplementary Fig. 11. Posterior predicted time (95% credibility interval) the start of infection to three anti-spike IgG thresholds (42, 28, and 6 ng ml^{-1}) by age (20, 40, 60, 80 years), sex, and ethnicity from the multivariable linear model in 3271 participants. a, Time from the start of infection to the positivity threshold of 42 ng ml^{-1}. **b,** Time from the start of infection to the equivocal threshold of 28 ng ml^{-1}, which corresponds to 50% protection against PCR-confirmed reinfection. **c**, Time from the start of infection to 6 ng ml^{-1}, which corresponds to 50% protection against severe infection. **d**, Time from the start of infection to the above three thresholds multiplied by 2, 3, 5, and 10, in a 60-year-old white male as an example, to estimate the duration given the higher antibody level required for protection against variants of concern. Estimations are shown in **Supplementary Table 8**.
