# Supplementary material for: Anti-spike antibody response to natural SARS-CoV-2 infection in the general population
Source: Nat Commun. 2021 Oct 29;12:6250. doi: 10.1038/s41467-021-26479-2 (PMC8556331; doi:10.1038/s41467-021-26479-2)
Supplement: Supplementary file 3 — Reporting Summary [file 41467_2021_26479_MOESM3_ESM.pdf]

nature research

Corresponding author(s): David Eyre

Last updated by author(s): Oct 4, 2021

Reporting Summary

Nature Research wishes to improve the reproducibility of the work that we publish. This form provides structure for consistency and transparency in reporting. For further information on Nature Research policies, see our Editorial Policies and the Editorial Policy Checklist.

Statistics

For all statistical analyses, confirm that the following items are present in the figure legend, table legend, main text, or Methods section.

n/a	Confirmed	
☐	☒	The exact sample size (*n*) for each experimental group/condition, given as a discrete number and unit of measurement
☐	☒	A statement on whether measurements were taken from distinct samples or whether the same sample was measured repeatedly
☐	☒	The statistical test(s) used AND whether they are one- or two-sided<br>*Only common tests should be described solely by name; describe more complex techniques in the Methods section.*
☐	☒	A description of all covariates tested
☐	☒	A description of any assumptions or corrections, such as tests of normality and adjustment for multiple comparisons
☐	☒	A full description of the statistical parameters including central tendency (e.g. means) or other basic estimates (e.g. regression coefficient) AND variation (e.g. standard deviation) or associated estimates of uncertainty (e.g. confidence intervals)
☐	☒	For null hypothesis testing, the test statistic (e.g. *F*, *t*, *r*) with confidence intervals, effect sizes, degrees of freedom and *P* value noted<br>*Give P values as exact values whenever suitable.*
☐	☒	For Bayesian analysis, information on the choice of priors and Markov chain Monte Carlo settings
☒	☐	For hierarchical and complex designs, identification of the appropriate level for tests and full reporting of outcomes
☐	☒	Estimates of effect sizes (e.g. Cohen's *d*, Pearson's *r*), indicating how they were calculated

Our web collection on statistics for biologists contains articles on many of the points above.

Software and code

Policy information about availability of computer code

Data collection	De-identified study data were accessed through the Office for National Statistics (ONS) Secure Research Service (SRS). The data available in SRS were prepared for data analysis using Stata MP 16.
Data analysis	All analyses were performed in R 3.6 using the following packages: tidyverse (version 1.3.0), brms (version 2.14.0), rstanarm (version 2.21.1), splines (version 3.6.1), lcmm (version 1.9.2), nnet (version 7.3-14), ggeffects (version 0.14.3), arsenal (version 3.4.0), cowplot (version 1.1.0), bayesplot (version 1.7.2). A copy of the analysis code is available at https://github.com/jiaweioxford/COVID19_infection_antibody_response.

For manuscripts utilizing custom algorithms or software that are central to the research but not yet described in published literature, software must be made available to editors and reviewers. We strongly encourage code deposition in a community repository (e.g. GitHub). See the Nature Research guidelines for submitting code & software for further information.

Data

Policy information about availability of data

All manuscripts must include a data availability statement. This statement should provide the following information, where applicable:

- Accession codes, unique identifiers, or web links for publicly available datasets
- A list of figures that have associated raw data
- A description of any restrictions on data availability

De-identified study data are available for access by accredited researchers in the ONS Secure Research Service (SRS) for accredited research purposes under part 5, chapter 5 of the Digital Economy Act 2017. Individuals can apply to be an accredited researcher using the short form on https://researchaccreditationservice.ons.gov.uk/ons/ONS_registration.ofml. Accreditation requires completion of a short free course on accessing the SRS. To request access to data in the SRS, researchers must submit a research project application for accreditation in the Research Accreditation Service (RAS). Research project applications are considered by the project team and the Research Accreditation Panel (RAP) established by the UK Statistics Authority at regular meetings. Project

application example guidance and an exemplar of a research project application are available. A complete record of accredited researchers and their projects is published on the UK Statistics Authority website to ensure transparency of access to research data. For further information about accreditation, contact Research.Support@ons.gov.uk or visit the SRS website.

Field-specific reporting

Please select the one below that is the best fit for your research. If you are not sure, read the appropriate sections before making your selection.

☒ Life sciences ☐ Behavioural & social sciences ☐ Ecological, evolutionary & environmental sciences

For a reference copy of the document with all sections, see nature.com/documents/nr-reporting-summary-flat.pdf

Life sciences study design

All studies must disclose on these points even when the disclosure is negative.

Sample size	7,256 participants aged ≥16 years from the general population of the United Kingdom who had a swab PCR-confirmed infection episode between 26 April 2020 and 14 June 2021 contributed a total of 14,552 SARS-CoV-2 anti-spike IgG measurements taken at any point between 90 days before the index positive date through to 180 days after. All available data were used for the current study, with the timing of the analysis determined by the duration of follow up available, rather than sample size given the number of participants in the study.
Data exclusions	No available data were excluded from the study.
Replication	All measurements and analytical assays were undertaken once given the scale of the study and cost limitations with 100,000s of assays performed. Serially collected samples from the same participants included demonstrate reproducibility over time.<br>The statistical analyses have been successfully replicated by two individuals.
Randomization	Recruitment randomised - we used data from the UK's Office for National Statistics (ONS) COVID-19 Infection Survey (CIS) (ISRCTN21086382) which randomly selects private households on a continuous basis from address lists and previous surveys conducted by the ONS or the Northern Ireland Statistics and Research Agency to provide a representative sample across the four countries comprising the UK (England, Wales, Northern Ireland, Scotland). No intervention.
Blinding	Not done. This was an observational study with no interventions. Results were returned to participants to support their involvement, but this would not be expected to impact the study findings.

Reporting for specific materials, systems and methods

We require information from authors about some types of materials, experimental systems and methods used in many studies. Here, indicate whether each material, system or method listed is relevant to your study. If you are not sure if a list item applies to your research, read the appropriate section before selecting a response.

Materials & experimental systems

n/a	Involved in the study	
☐	☒	Antibodies
☒	☐	Eukaryotic cell lines
☒	☐	Palaeontology and archaeology
☒	☐	Animals and other organisms
☐	☒	Human research participants
☐	☒	Clinical data
☒	☐	Dual use research of concern

Methods

n/a	Involved in the study	
☒	☐	ChIP-seq
☒	☐	Flow cytometry
☒	☐	MRI-based neuroimaging

Antibodies

Antibodies used	The calibrant (mAb45) provided as part of the Thermo Fisher OmniPATH 384 Combi SARS-CoV-2 IgG ELISA kit is a monoclonal antibody. It is available as part of the test kit. CR3022 and mAb269 were produced at the University of Oxford, details on availability can be provided by the authors on request.
Validation	Details of the validation of the the Thermo Fisher OmniPATH 384 Combi SARS-CoV-2 IgG ELISA kit are provided in the manufacturer's instructions for use. The assay has also been validated in a head-to-head comparison of similar assays (https://doi.org/10.1016/S1473-3099(20)30634-4), where the sensitivity was 99·1% (95%CI 97·8–99·7) and specificity was 99·0% (98·1–99·5). The assay calibrant, mAb45, is described in https://doi.org/10.1016/10.1016/j.cell.2021.02.032.<br><br>The CR3022 monoclonal antibody is described and validated in https://doi.org/10.1016/j.chom.2020.06.010. The mAb269 is described and validated in https://doi.org/10.1016/j.cell.2021.02.033.

Further validation of the antibodies used was performed by comparing serial dilution series of these antibodies on the test platform, ensuring a sigmoidal dose response that these saturated at the upper limit of detection. Reproducibility between batches and stability over time was also assessed using the same method. Comparisons of the performance of these antibodies on a commercial anti-S antibody detection platform are provided in https://doi.org/10.1016/j.cmi.2021.05.041.

Human research participants

Policy information about studies involving human research participants

Population characteristics	The median (IQR) age was 47 (34-59) years and 3,874 (53.4%) were female. 6,577 (90.6%) were of white ethnicity. 127 (1.8%) were healthcare workers, and 1,592 (21.9%) had a long-term health condition.
Recruitment	The Office for National Statistics (ONS) COVID-19 Infection Survey (CIS) is a large household survey with longitudinal follow-up (ISRCTN21086382, https://www.ndm.ox.ac.uk/covid-19/covid-19-infection-survey/protocol-and-information-sheets) (details in20). The study received ethical approval from the South Central Berkshire B Research Ethics Committee (20/SC/0195). Private households are randomly selected on a continuous basis from address lists and previous surveys to provide a representative sample across the UK. Following verbal agreement to participate, a study worker visited each selected household to take written informed consent for individuals aged 2 years and over. Parents or carers provided consent for those aged 2-15 years; those aged 10-15 years also provided written assent. To reduce the probability that invited individuals/households decide not to respond to the survey they are paid generously to participate in the survey, see https://www.ons.gov.uk/surveys/informationforhouseholdsandindividuals/householdandindividualsurveys/covid19infectionsurveycis/howtotakepart. All participants who completed the enrolment visit was offered a £50 voucher, and one £25 voucher for each further visit. For the current analysis we only included individuals aged 16 years and over.<br><br>Nevertheless a certain degree of non-response is inevitable. While certain factors might drive non-response to invitations to participate, adjustment for covariates that may influence selection into the sample ensures that estimates of relative effects are not biased by factors that both influence selection into the sample and the risk of the outcome (model-based inference). Factors that were included in the models are listed below. We cannot exclude the possibility that other unmeasured factors that could influence self-selection into the survey and are not strongly associated with factors already included in the model could bias the result.<br><br>When fitting latent class models to antibody responses we adjust for age, reported long-term health conditions, Ct values, and self-reported symptoms as covariates for class membership. When fitting the Bayesian linear mixed models, we adjust for age, sex, ethnicity, reported long-term health conditions, Ct values, and self-reported symptoms as covariates in the multivariable model.
Ethics oversight	The study received ethical approval from the South Central Berkshire B Research Ethics Committee (20/SC/0195).

Note that full information on the approval of the study protocol must also be provided in the manuscript.

Clinical data

Policy information about clinical studies

All manuscripts should comply with the ICMJEguidelines for publication of clinical research and a completedCONSORT checklist must be included with all submissions.

Clinical trial registration	ISRCTN21086382
Study protocol	https://www.ndm.ox.ac.uk/covid-19/covid-19-infection-survey/protocol-and-information-sheets
Data collection	We used data from the UK's Office for National Statistics (ONS) COVID-19 Infection Survey (CIS) (ISRCTN21086382) from 26 April 2020 to 14 June 2021.
Outcomes	SARS-CoV-2 antibody levels were measured using an ELISA detecting anti-trimeric spike IgG developed by the University of Oxford (Thermo Fisher OmniPATH 384 Combi SARS-CoV-2 IgG ELISA).<br><br>For individuals infected with SARS-CoV-2, we identified non-responders who did not seroconvert using latent class mixed model, and estimated the peak levels and half-life of anti-spike IgG antibody response post natural infection among those who seroconverted using Bayesian linear mixed effects model.<br><br>Secondary analyses included:<br>a) Determining associations between seronegative non-responders with demographic factors and individual symptoms using logistic regression.<br>b) Examining the impacts of age, sex, ethnicity, reported long-term health conditions, Ct values, and self-reported symptoms on anti-spike IgG antibody peak levels and half-life using multivariable Bayesian linear mixed effects model.<br>c) Estimating the duration of protection from infection to the positivity threshold (42 ng/ml), the threshold associated with 50% protection against reinfection (28 ng/ml), and the threshold associated with 50% protection against severe infection (6 ng/ml) using predictions from the Bayesian linear mixed effects model.
